# Recent Advances in Genetic Technique of Microbial Report Cells and Their Applications in Cell Arrays

**DOI:** 10.1155/2015/182107

**Published:** 2015-09-07

**Authors:** Do Hyun Kim, Moon Il Kim, Hyun Gyu Park

**Affiliations:** ^1^Department of Bionano Technology, Gachon University, Gyeonggi 461-701, Republic of Korea; ^2^Department of Chemical and Biomolecular Engineering (BK21+ Program), Korea Advanced Institute of Science and Technology (KAIST), Daejeon 305-701, Republic of Korea

## Abstract

Microbial cell arrays have attracted consistent attention for their ability to provide unique global data on target analytes at low cost, their capacity for readily detectable and robust cell growth in diverse environments, their high degree of convenience, and their capacity for multiplexing via incorporation of molecularly tailored reporter cells. To highlight recent progress in the field of microbial cell arrays, this review discusses research on genetic engineering of reporter cells, technologies for patterning live cells on solid surfaces, cellular immobilization in different polymers, and studies on their application in environmental monitoring, disease diagnostics, and other related fields. On the basis of these results, we discuss current challenges and future prospects for novel microbial cell arrays, which show promise for use as potent tools for unraveling complex biological processes.

## 1. Introduction

Biosensors, which have been widely utilized for the detection of target molecules, are increasingly important, owing to their broad applications in biotechnology and related fields, including disease diagnostics, environmental monitoring, drug discovery, and food processing [1]. Biosensor is operated mainly based on the specificity of biologically active molecules, including nucleic acids, enzymes, antibodies and antigens, receptors, and cells, and thus these substances are fundamental for the specific recognition of target molecules.

Among biosensors, cell-based or whole-cell biosensors have garnered particular interest because they can provide unique data on the global activities of test samples, such as their toxicity, genotoxicity, or bioavailability, from a direct assay on live cells [2]. The use of live cells also allows for reagent-free, nondestructive real-time monitoring of the biological effects as they develop, with no need for the pre- or posttreatment steps that are generally required for conventional chemistry-based analytical methods. Therefore, the use of whole cells as sensing entities can conveniently provide a diverse array of data on integrated biological effects that cannot be achieved using other biosensors, although the relative insufficiency of their specificity is inevitable, based on the nature of living systems.

Previous cell-based biosensors typically used a single type cells, with a single function, to analyze a single sample; however, recent approaches have focused on the development of arrays comprised of multiple cells on a mapped solid surface that are subsequently exposed to mixtures containing multicomponent analytes, which can provide multiplexed output signals corresponding to the amount of each target in the sample mixture [3]. Compared to singleplex cell-based biosensors, cell arrays enable simultaneous detection of multiple samples with multiple output signals that can be used to rapidly analyze large numbers of samples. Due to the increased need for multiplex, high-throughput analysis capability, cell array techniques have recently garnered significant attention [4].

Diverse types of cell arrays have been reported. In particular, human cells have been extensively employed to develop miniaturized cell-based assay platforms, which include microfluidic and microarray biochips to mimic human metabolism [5–9]. These biochips have been extensively used in drug discovery to evaluate toxicity and other metabolic activities during adsorption, distribution, metabolism, and elimination of drug candidates in the human body [5]. Other eukaryotic cells, including yeast, have also been examined in applications for gene function analysis, microphysiometry, and therapeutic agent identification based on array platforms prepared using diverse microfabrication strategies such as photolithography, inkjet printing, or microcontact printing [10–17]. Due to the critical need for high-throughput methods for investigating the bioactivity of eukaryotic cells, cell arrays based on eukaryotic cells have come into widespread use.

**Figure 1:**
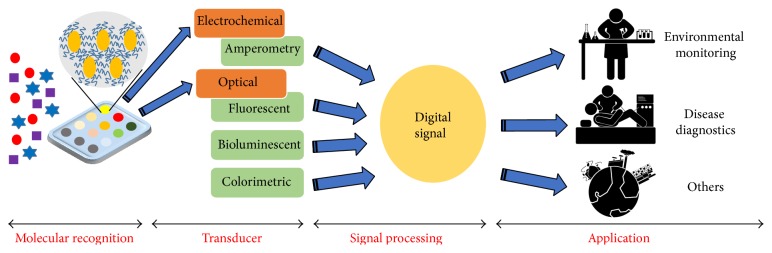
A schematic representation of a microbial cell array.

In contrast, arrays using prokaryotic cells have numerous differentiated benefits [18]. It is easy to grow and to maintain the viability of prokaryotic cells at low cost, large and homogeneous populations are easily obtainable, they are robust to a variety of physical and chemical environments, and they show low susceptibility to biological contamination. Furthermore, prokaryotic cells are amenable to physical or chemical manipulation, particularly those required for patterning in an array format. Perhaps their most important characteristic, based on recent developments in genetic engineering technology, is that prokaryotic cells can be molecularly engineered to respond in a dose-dependent manner, to yield readily quantifiable optical (colorimetric, fluorescent, or luminescent) or electrochemical signals to predetermined targets such as chemicals, biomolecules, or biological effects [3]. This is generally achieved by the fusion of a sensing element, a selective promoter along with its regulatory elements, to a suitable molecular reporter system. Moreover, advances in genetic engineering also allow the expression of two independent reporter systems in a single microorganism, facilitating multiplex analysis and particular logic operations of microbial cell arrays [19–21]. These genetically engineered sensor cells are patterned on a solid surface, incorporated into a single hardware platform, and simultaneously exposed to a sample, for applications such as environmental monitoring, disease diagnostics, and others (Figure 1). Due to the significance and widespread applicability of this state-of-the-art technology, it has garnered increasing public attention, and a reasonable research direction must be set to widely expand its utilization for both laboratory and field use.

In this paper, we review recent advances in microbial cell arrays. Recent research into genetic engineering of reporter cells, technologies for patterning live cells on solid surfaces, and their immobilization in different polymers are extensively discussed, along with studies of related applications. We present the current challenges and future prospects for novel microbial cell arrays, which can be used as potent tools for unraveling complex biological processes.

## 2. Genetic Engineering of Microbial Reporter Cells

Although unmodified bacteria have been used as biosensors, based on changes in natural bioluminescence as cells grow, genetic engineering of microbial cells is extensively employed to rationally produce dose-dependent signals to predetermined environmental stimuli [22, 23]. Typical engineering methods include fusion of a reporter gene system to promoters from selected stress-response regions, resulting in specific cell growth and easily measurable signals that are proportional to the quantity of target analytes, including chemicals, nutrients, or heavy metals. To date, fluorescence- and luminescence-based signals have typically been produced in microbial cell arrays by the activity of corresponding reporter genes that express fluorescent protein and bacterial luciferase (lux), respectively [24, 25]. In recent years, several attempts have been made to improve the performance of reporter cells, such as further engineering of regulatory regions, splitting of the lux operon, increasing cellular permeability, or shuffling of gene elements [26–31].

Recently, another class of genetic engineering, based on construction of mutant bacteria with auxotrophic characteristics, has garnered attention, owing to its capacity to specifically and sensitively detect diverse types of metabolites present in the metabolic pathways of the microbial cells on the array [32, 33]. Several different strategies, such as transposon or N-methyl-N′-nitro-N-nitrosoguanidine- (NTG-) induced mutagenesis and chromosomal gene deletion based on linear cassettes, have been employed to prepare auxotrophic bacteria [34, 35]. Bioluminescence-producing firefly luciferase or fluorescent protein reporter genes have also been used to produce corresponding optical responses that are proportional to the concentrations of target analytes. These cell arrays were proven to enable rapid (<4 h) and simultaneous analysis of multiple targets from complex biological fluids [33, 36].

As described above, the signals generated by the arrays have primarily been produced by proteins or by the activity of an enzyme expressed using a reporter gene system. Depending on the type of reporter gene, the signals emitted have been detected optically or electrochemically. Additional signaling methods based on commercially available Live/Dead staining or surface plasmon resonance analysis have also been reported, for diversification of cell array detection mechanisms [37, 38].

## 3. Patterning Microbial Cells on Solid Surfaces

In addition to cell arrays patterned in the wells of premade microtiter plates, target microbial cells can be patterned on a solid surface, to maximize the number of cell spots per unit area while enabling the activity of each spot to be distinguished from that of its neighbors, without cross-contamination. Diverse microfabrication strategies, such as photolithography, soft lithography, and noncontact printing, have been employed to prepare patterned cell arrays on numerous materials such as silicon, glass, various polymers, and gold [38–44].

Photography-based processes have been widely applied to prepare patterns of immobilized bacterial cells. Typically, a water-soluble photoresist polymer is employed for the fabrication of a three-dimensional matrix on the desired region by simple exposure to UV light, resulting in accommodation of both target cells and culture medium in the matrix. Using this strategy, silicon chips consisting of microfluidic channels, microchambers, valves, and additional structures have successfully been prepared for toxicity monitoring, based on generation of* Escherichia coli* microspots on a planar array [40].

Microcontact printing, one of the most conventionally used soft lithography methods to prepare patterns with a chemical moiety, has been employed to create cellular patterns on both planar and nonplanar surfaces, by delivering anchor molecules using a polydimethylsiloxane (PDMS) stamp [45]. Using this stamp, self-assembled monolayers, which can adsorb to patterned gold surfaces, form covalent bonds with a protein that guides a cell to the pattern. Using this strategy, high-resolution printing of massive arrays of various microorganisms, such as* Lactobacillus plantarum*,* E. coli*,* Candida albicans*, and fungal spores of* Aspergillus fumigatus*, has been reported on porous aluminum oxide [46]. Another bacterial array, based on a combination of self-assembled polyelectrolyte multilayers and micromolded poly(ethylene glycol)-poly(lactide) diblock copolymers to promote target cell adhesion, has also been reported [47].

As an example of noncontact printing, piezoelectric inkjet printers have been used to prepare high-density live cell arrays for screening antimicrobial activity [48]. Flickinger et al. reported the formulation of reactive microbial inks and the use of piezo tips to spot* E. coli* at designated positions [49]. A noncontact robotic printer was also employed to prepare* E. coli* arrays with several nanoliter-volume spots on chemically modified glass [50].

## 4. Maintenance of Cell Viability

For practical application of microbial cell arrays, cells on the array should maintain their viability and be able to be stored for sufficiently long periods of time. Thus, development of efficient solid-phase arrays by appropriate immobilization of cells has garnered consistent attention, particularly in industry. Various polymers, such as agar, agarose, alginate, collagen, latex, polyacrylamide, polyethylene glycol diacrylate, and carrageenan, as well as freeze/vacuum drying, have been reported to immobilize cells while retaining sufficient viability [25, 33, 43, 49, 51–56]. In particular, further addition of components such as glycerol or trehalose was shown to effectively provide extracellular or intracellular protection and thus to enhance the long-term survival rate [50, 57]. Vacuum drying of As(III) reporter bacteria in the presence of 34% trehalose and 1.5% polyvinylpyrrolidone resulted in very effective preservation of initial activity during up to 12 weeks of storage at 4°C [58]. An innovative strategy based on the formation of bacterial spores was also reported for long-term (up to 2 years) preservation of sensing cells at room temperature [59].

## 5. Applications of Microbial Cell Arrays

Based on their abovementioned characteristics, microbial cell arrays have been used in diverse applications for monitoring the global effects of test samples, as shown briefly in Table 1. In this section, we describe recent studies of the application of microbial cell arrays, categorized by environmental monitoring, disease diagnostics, and others.

### 5.1. Environmental Monitoring

Although the envisaged applications of microbial cell array are numerous, they have primary been applied in the environmental field. Microbial cells have been elaborately modified to produce both qualitative and quantitative outputs in response to single or multiple kinds of environmental stimuli and applied to construct cell arrays to analyze multiple test samples. Due to their capacity to show the unique responses of live cells, microbial cell arrays can serve as a potent analytical route to replace the conventional yet laborious methods currently in use.

Several microbial cell arrays have been developed for assaying heavy metals, which are regarded as major toxic elements. Biran et al. reported a microbial cell array that employs a genetically engineered* E. coli* strain that contains the* lacZ* reporter gene, which can express *β*-galactosidase, fused to a the promoter of a heavy metal-responsive gene. A plasmid carrying the gene coding for the enhanced cyan fluorescent protein was subsequently introduced into this sensing strain to produce concomitant optical signals in proportion to the quantity of the target heavy metal, mercury. Levels as low as 100 nM Hg^2+^ could be detected after only 1 h of incubation [60]. Arsenic and cadmium were also simultaneously quantified via a multichannel bioluminescent* E. coli* array system, although cross-reactivity was observed when the two metals were mixed [61].

Other environmental pollutants have also been monitored using microbial cell arrays. Gou et al. utilized a green fluorescent protein-fused recombinant* E. coli* array to assess the mechanistic toxicity of silver and titanium oxide nanoparticles by measuring real-time gene expression profiles [62]. A portable biosensor device, based on engineered yeast and bacterial cells fused to a reporter gene expressing luciferase, was reported to be able to detect several endocrine disruptors, including androgens and estrogens [53]. Ahn et al. reported an* E. coli* array consisting of optically coded functional microbeads containing both bioluminescent reporter bacterial cells and fluorescent microspheres for broad-range toxicity monitoring [63]. A bacterial cell array using recombinant* E. coli* in 384-well plates was also employed in a genome-wide investigation of the toxic mechanisms of naphthenic acids, chemicals that pose serious environmental hazards and which are present in effluents from petrochemical processing [64]. Three different chemicals that cause either superoxide damage (paraquat), DNA damage (mitomycin C), or protein/membrane damage (salicylic acid) were also successfully detected within 2 h, using a bacterial cell array based on bioluminescent* E. coli* [25].

### 5.2. Disease Diagnostics

Recently, cell-based assays employing fast-growing auxotrophic bacteria supplemented with bioluminescent or fluorescent reporter genes have been shown to rapidly, conveniently, and simultaneously detect multiple target molecules relevant to human diseases. In contrast to conventional diagnostic methods, which often require numerous experimental steps or complicated and expensive instrumentation, bacterial auxotroph-based arrays show rapid, specific, and sensitive cell growth in direct response to the concentration of the corresponding molecules. This method can also be extended to evaluate or monitor nutritional conditions, as the metabolic pathways of microbial cells contain many relevant metabolites.

Several cell-based approaches to diagnosis of human diseases have been reported. A multiplexed amino acid array for simultaneously quantifying 16 different amino acids based on the rapid and specific growth of amino acid-auxotrophic* E. coli* was reported [33]. Using this array, multiple amino acids in biological fluids were quantitatively determined within 4 h, simply by measuring bioluminescent signals from immobilized cells, without any pre- or posttreatment. Using this system, two different kinds of metabolic diseases of newborn babies, phenylketonuria and homocystinuria, were successfully diagnosed by measuring luminescence values from phenylalanine and methionine auxotrophs incubated with an eluted mixture from clinical blood paper specimens. Similarly, homocysteine, an important marker for cardiovascular disease and other syndromes such as Alzheimer's and Parkinson's disease, neural tube defects, pregnancy complications, and osteoporosis, was quantified by employing another bioluminescent* E. coli* array, which showed high specificity, sensitivity, and excellent levels of precision and reproducibility [65]. Galactosemia, a major metabolic disorder of newborns, was also successfully diagnosed by employing* galT*-knockout* E. coli* [66]. Furthermore, simultaneous quantification of multiple amino acids in a single biological sample was reported and applied in the multiplexed diagnosis of three key metabolic diseases of newborn babies [36]. The assay utilized three* E. coli* auxotrophs that grow only in the presence of the corresponding target amino acids and contain three different fluorescent reporter plasmids that produce distinguishable fluorescence signals (red, green, and cyan) in concert with cell growth. The three auxotrophs were mixed and immobilized in the same well of a 96-well plate and consequently yielded three different fluorescence signals that corresponded to the reporter plasmids. The clinical utility of this assay system was demonstrated by employing it to identify metabolic diseases of newborns through the quantification of phenylalanine, methionine, and leucine in clinically derived dried blood specimens.

### 5.3. Others

Based on their unique advantages, the applications for microbial cell arrays are currently being expanded. Held et al. reported a bacterial cell array with an automated flow-injection system for the selective and simultaneous determination of various mono- and disaccharides [55]. The selectivity of the array was achieved by combination of the metabolic responses of* E. coli* mutants lacking different transport systems for individual carbohydrates. The array enabled simultaneous detection of three major sugars, fructose, glucose, and sucrose, in test samples. A unique array of bacterial colonies has been reported for large-scale gene expression analysis [67]. In this system, recombinant* E. coli* clones containing plasmid-encoded copies of several thousand individually expressed sequence tags were spotted and incubated for ~6 h to allow bacterial growth and consequent amplification of the cloned tags. For use in drug discovery, an array of* Staphylococcus aureus* fused with* lux* (luciferase-producing) plasmids was reported for screening antibiotic activity [68]. Finally, a panel of 15 bioluminescent* E. coli* containing multiple bacterial reporter genes associated with oxidative stress, DNA damage, heat shock, and efflux of excess metals was arrayed to screen a library of 420 pharmaceuticals [69]. This work demonstrated that microbial cell arrays can play a significant role in drug development alongside in vitro toxicity tests.

## 6. Conclusion and Future Prospects

As described above, microbial cell arrays have been widely investigated and have garnered significant attention as a potent analytical paradigm, due to their capacity to provide unique global data for live systems. The arrays provide the option, which was previously unavailable, of analyzing biological reactions via real-time monitoring of the responses of an unlimited number of genetically tailored sensor strains, which provide easily measurable, dose-dependent optical or electrical signals within a short period of time. However, several challenges that significantly hinder the widespread utilization of microbial cell arrays remain, such as their limited viability and biological function after long-term storage, insufficient specificity, limited types of target analytes, and problems in genetic engineering of sensor strains (Table 2). However, significant progress to overcome these limitations is continuously being made, as shown in some of the approaches reviewed here, and microbial cell arrays show great promise for an increasing number of applications in diverse fields such as environmental monitoring, disease diagnostics, and drug discovery.

For microbial cell arrays to be positioned as a next-generation analytical tool, the following technological hurdles must be overcome before the technology matures. Possibly the most urgent need for practical applications is a dramatic improvement in the maintenance of cell activity and viability over prolonged periods of time. Many different approaches have been suggested, such as appropriate immobilization of cells or addition of particular additives to reduce stress factors; however, other innovative methods to significantly extend the shelf life of arrays are required, particularly for commercialization in industry. In addition, further engineering of reporter cells for higher specificity, sensitivity, and robustness or better methodologies for incorporation of such cells into hardware platforms will also greatly contribute to the widespread utilization of this technology. When they mature, microbial cell arrays may become an efficient and practical analytical tool in diverse biotechnological fields.

In summary, this paper highlights recent progress in the field of microbial cell arrays. Although the relative insufficiency of their specificity is inevitable, based on the nature of living systems, microbial cell arrays can provide unique data on the global activity of test samples with a range of advantages that are not achievable using other analytical methods. Diverse technological advances have provided the tools, materials, and elaborately engineered reporter cells needed to construct highly integrated arrays. Based on the unique advantages and continued progress in this field, we believe that microbial cell arrays will lead a new wave of novel diagnostic methods in environmental monitoring, disease diagnostics, drug screening, and other related fields.

## Figures and Tables

**Table 1:** Recent applications of microbial cell arrays.

Application field	Target	Microorganism	Detectable output	References
Environmental monitoring	Mercury	Recombinant *Escherichia coli*	Fluorescence	[60]
Environmental monitoring	Cadmium and arsenic III	Recombinant *E*. *coli*	Bioluminescence	[61]
Environmental monitoring	Silver and titanium oxide nanoparticles	Recombinant *E. coli*	Gene expression profile	[62]
Environmental monitoring	Endocrine disruptors	Recombinant yeast and *E. coli*	Bioluminescence	[53]
Environmental monitoring	Cell-damaging stress	Recombinant *E. coli*	Bioluminescence	[63]
Environmental monitoring	Naphthenic acid	Recombinant *E. coli*	Fluorescence	[64]
Environmental monitoring	Paraquat, mitomycin C, and salicylic acid	Recombinant *E. coli*	Bioluminescence	[25]
Disease diagnostics	16 amino acids	Recombinant *E. coli* auxotroph	Bioluminescence	[33]
Disease diagnostics	Homocysteine	Recombinant *E. coli* auxotroph	Bioluminescence	[65]
Disease diagnostics	Galactose	Recombinant *E. coli* auxotroph	Bioluminescence	[66]
Disease diagnostics	Phenylalanine, methionine, and leucine	Recombinant *E. coli* auxotroph	Fluorescence	[36]
Carbohydrates detection	Mono- and disaccharides	Recombinant *E. coli*	O_2_ reduction	[55]
Gene expression analysis	Growth of *E. coli* colonies	Recombinant *E. coli*	Gene expression profile	[67]
Screening antibiotics	Antibiotic activity	*Staphylococcus aureus*	Bioluminescence	[68]
Screening pharmaceuticals	420 pharmaceuticals	Recombinant *E. coli*	Bioluminescence	[69]

**Table 2:** Representative advantages and challenges of microbial cell arrays.

Advantages	Challenges
(1) Analysis of global activities of target analyte	(1) Limited viability and biological function
(2) Low cost	(2) Insufficient specificity
(3) Analysis is more convenient than existing technologies	(3) Types of target analytes are limited
(4) Robust to reaction environments	(4) Genetic stability of engineered reporter cell system is low
(5) Simultaneous detection of multiple analytes	(5) Laws limiting the use of genetically modified organisms
(6) Real-time, in situ monitoring	(6) Slow diffusion in cell membranes
